# Supplementary figures and images for: Mapping the Role of AcrAB-TolC Efflux Pumps in the Evolution of Antibiotic Resistance Reveals Near-MIC Treatments Facilitate Resistance Acquisition
Source: mSphere. 2020 Dec 16;5(6):e01056-20. doi: 10.1128/mSphere.01056-20 (PMC7771234; doi:10.1128/mSphere.01056-20)

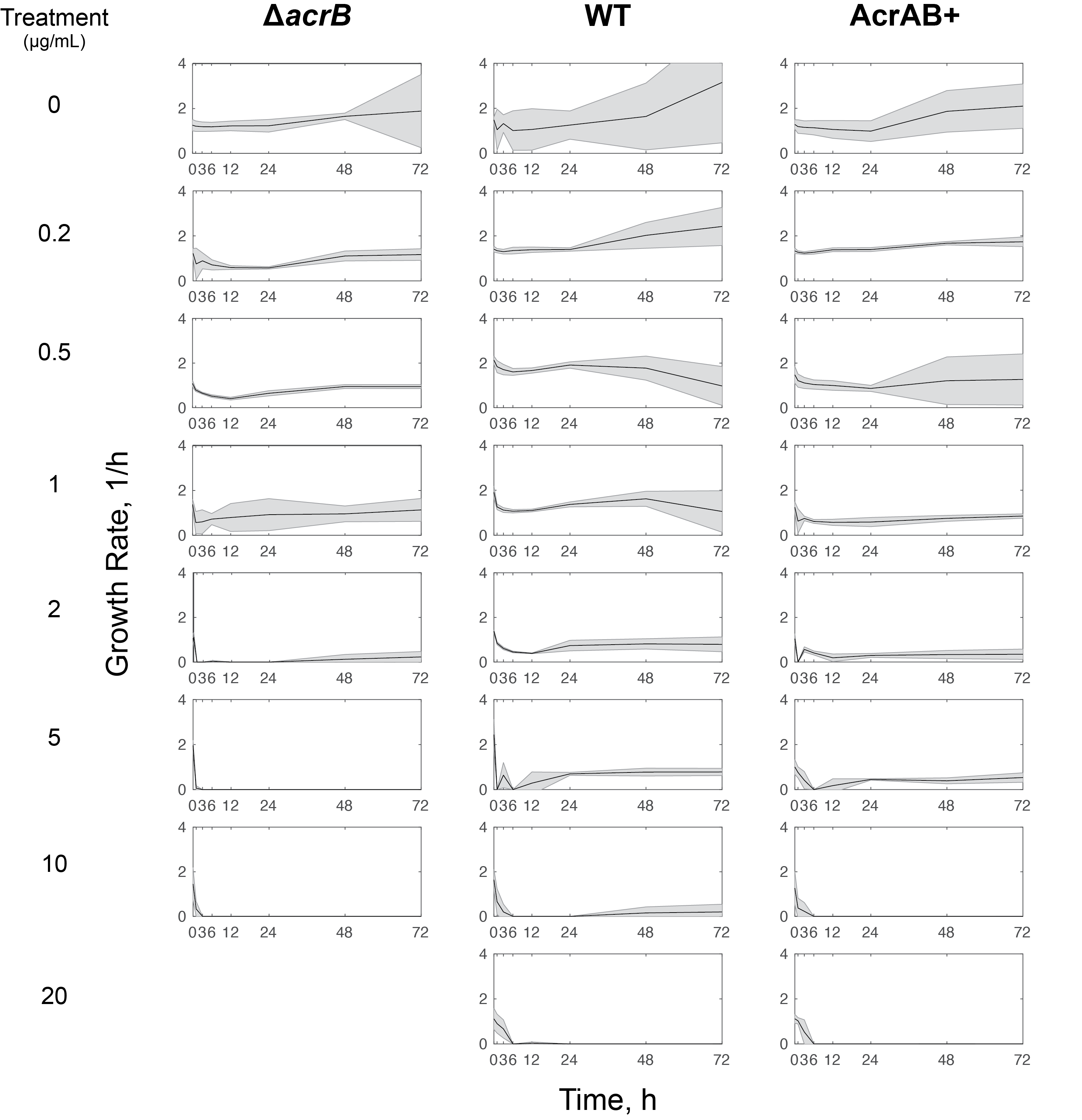

Supplement: FIG S1 [file mSphere.01056-20-sf001.tif]

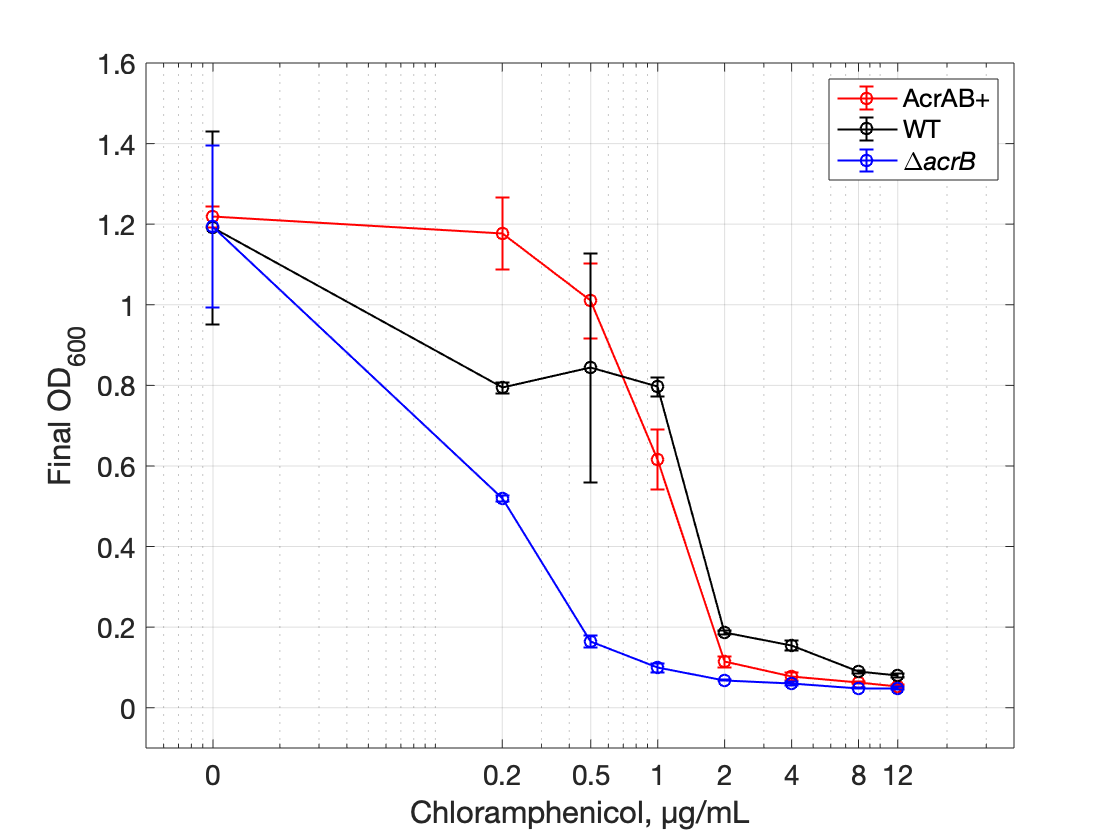

Supplement: FIG S2 [file mSphere.01056-20-sf002.tif]

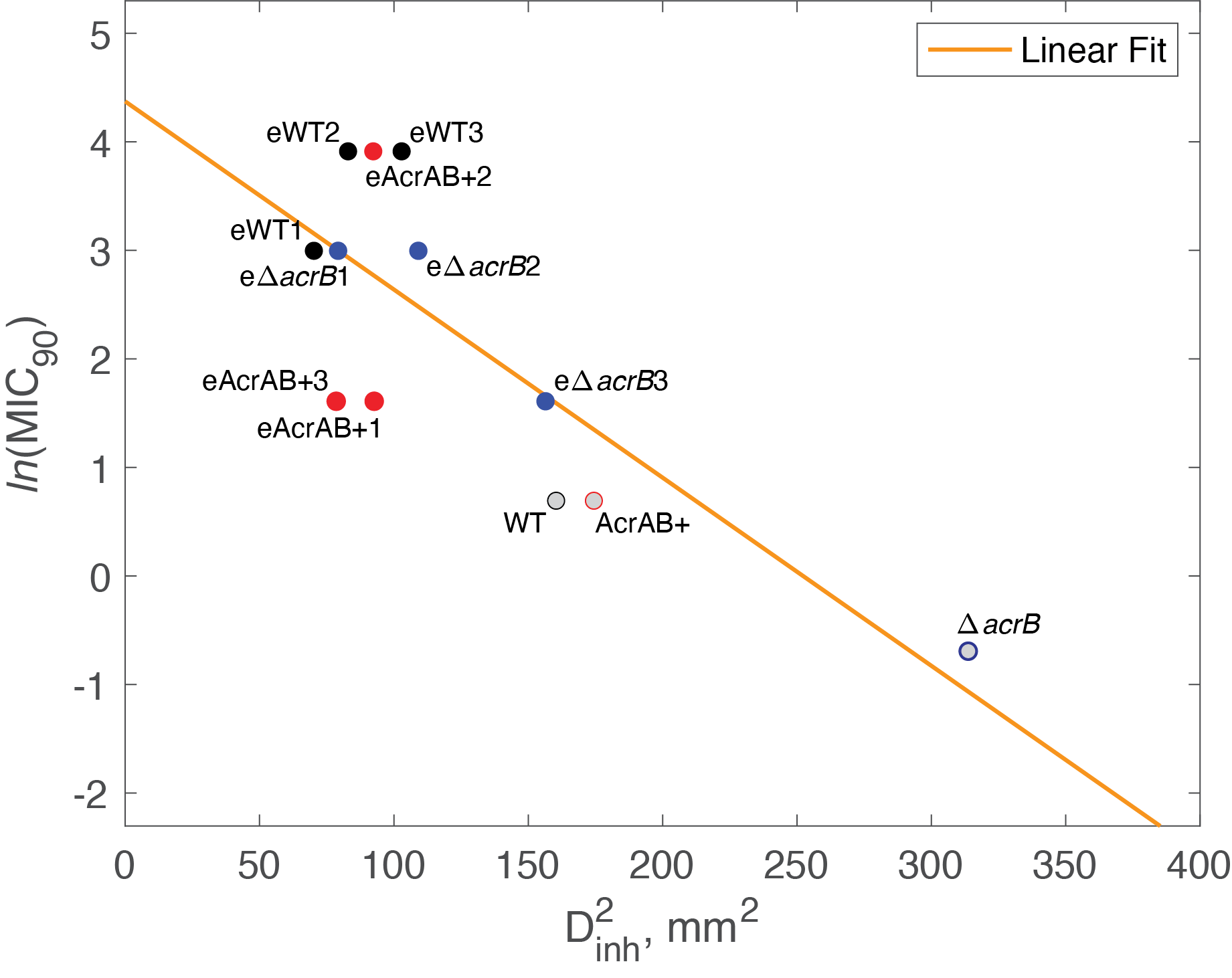

Supplement: FIG S5 [file mSphere.01056-20-sf005.tif]

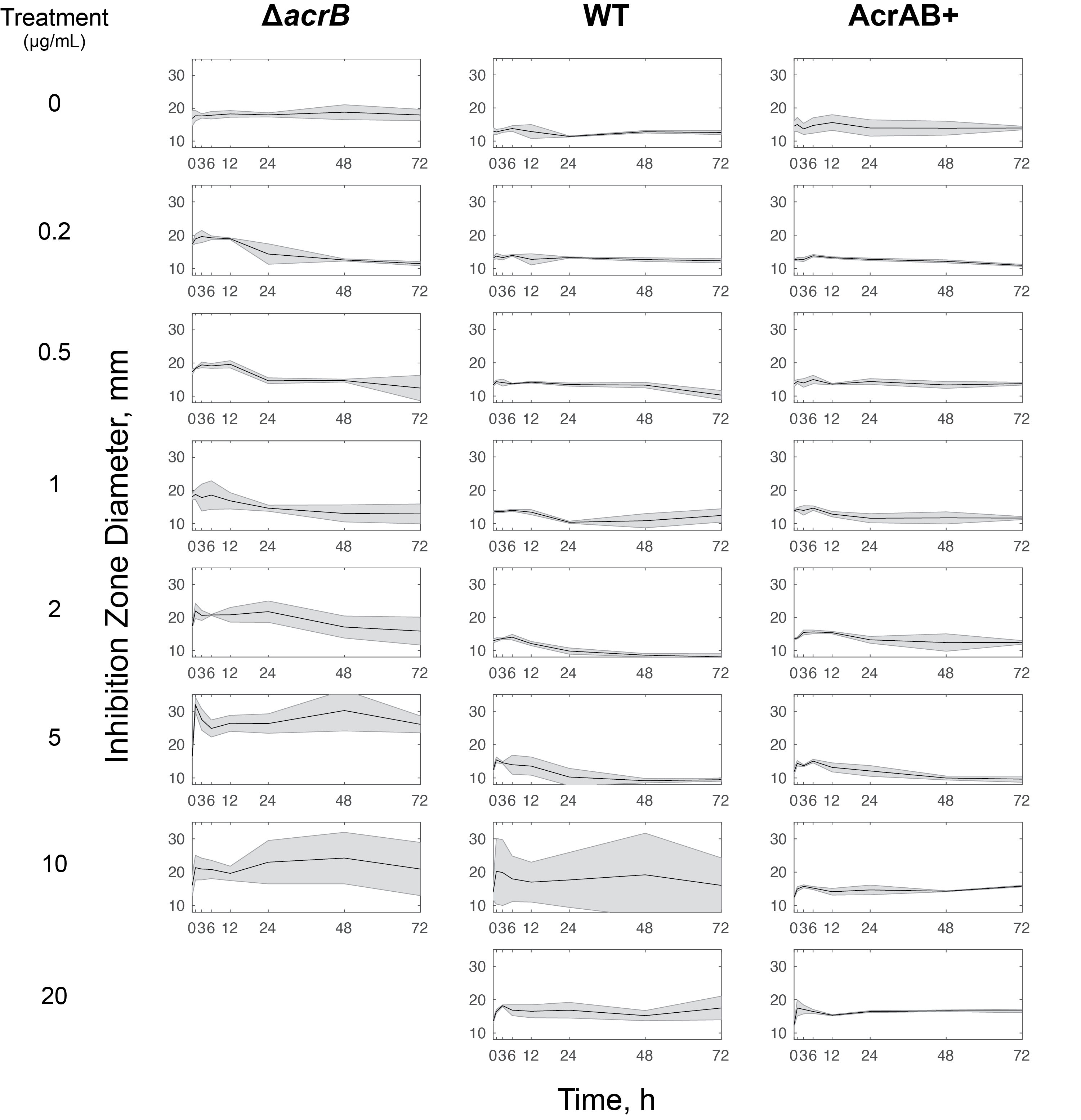

Supplement: FIG S3 [file mSphere.01056-20-sf003.tif]

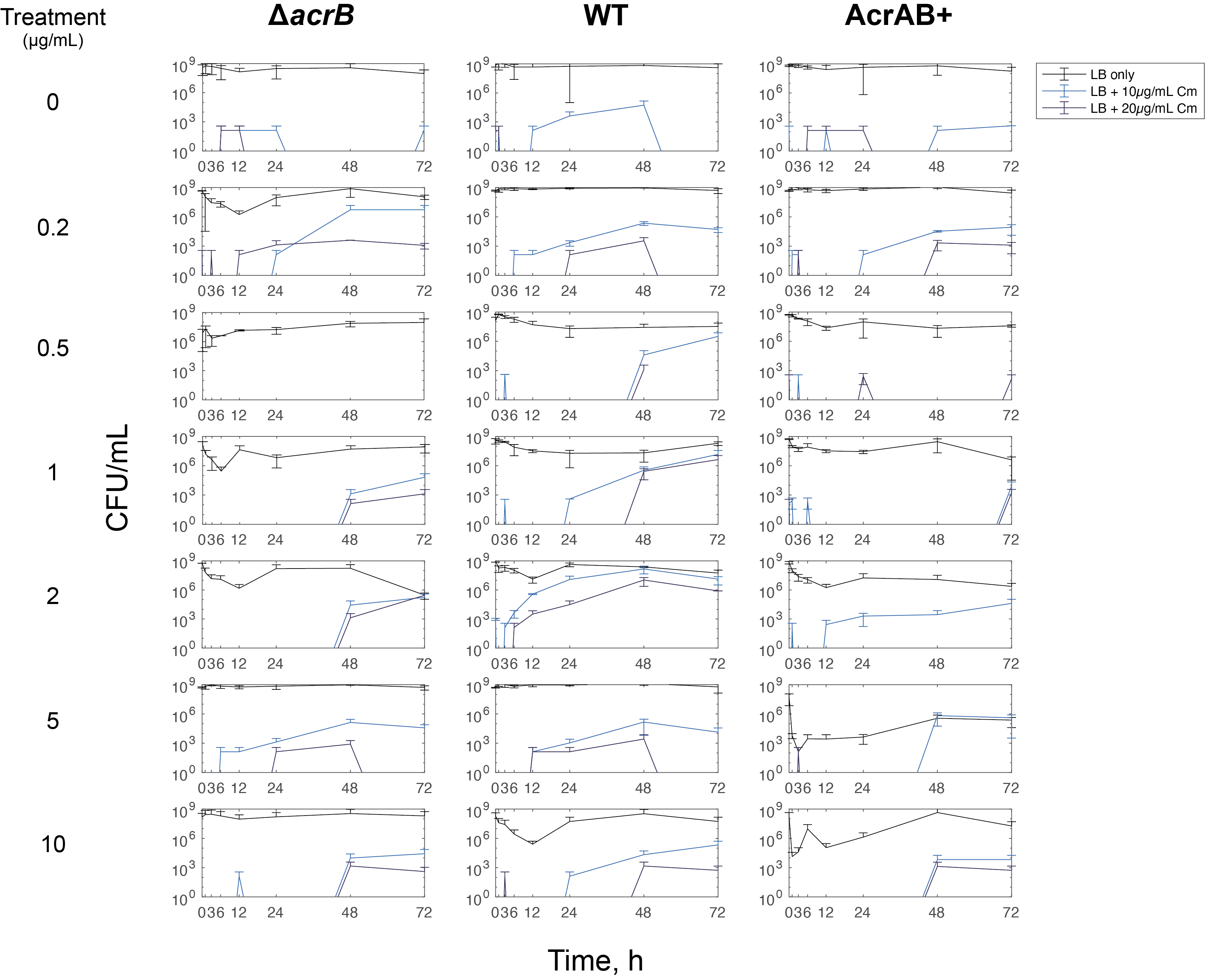

Supplement: FIG S4 [file mSphere.01056-20-sf004.tif]

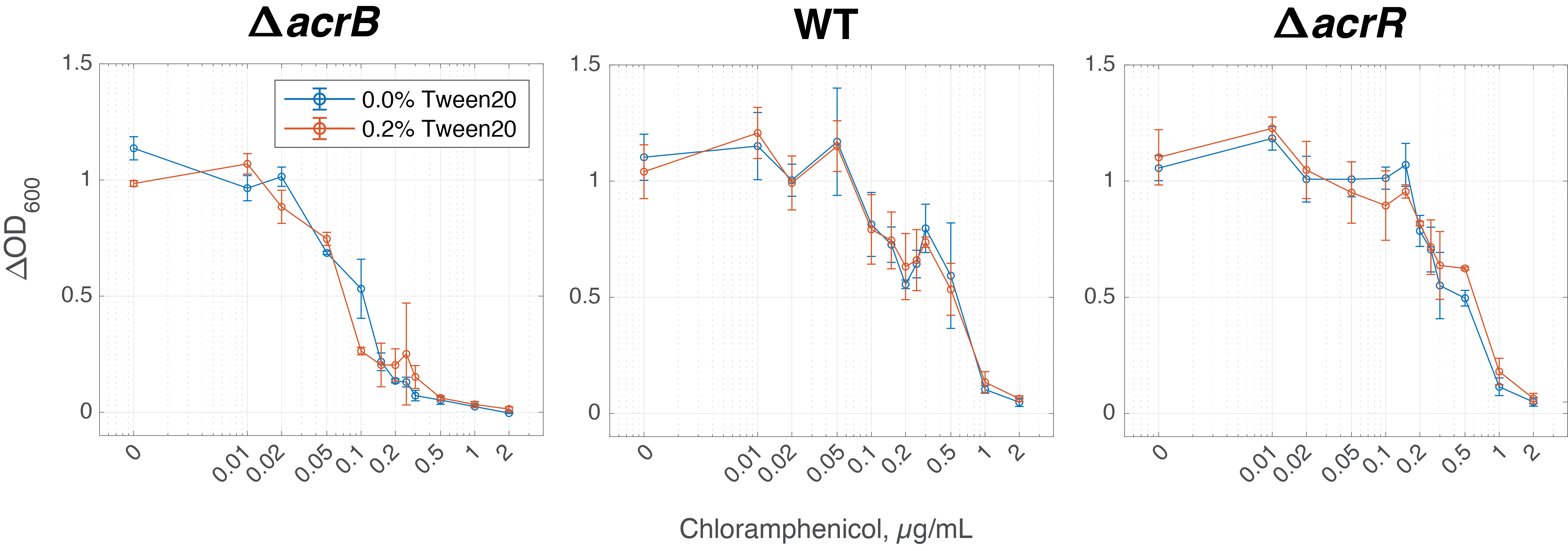

Supplement: FIG S6 [file mSphere.01056-20-sf006.tif]
