# Supplementary material for: Mapping the Role of AcrAB-TolC Efflux Pumps in the Evolution of Antibiotic Resistance Reveals Near-MIC Treatments Facilitate Resistance Acquisition
Source: mSphere. 2020 Dec 16;5(6):e01056-20. doi: 10.1128/mSphere.01056-20 (PMC7771234; doi:10.1128/mSphere.01056-20)
Supplement: TABLE S3 [file mSphere.01056-20-st003.docx]

| **Direction** | **Primer (5’ to 3’)** |
| --- | --- |
| Forward | ATGTATGTAAATCTAACGCCTGTAAATTCACGAACATATG**GTGTAGGCTGGAGCTGCTTC** |
| Reverse | CCTGGAGTCAGATTCAGGGTTATTCGTTAGTGGCAGGATT**GATCCGTCGACCTGCAGTT** |
